# First report of *Meloidogyne incognita* infecting *Cannabis sativa* in Alabama

**DOI:** 10.21307/jofnem-2021-052

**Published:** 2021-05-01

**Authors:** Bisho R. Lawaju, William Groover, Jessica Kelton, Kassie Conner, Edward Sikora, Kathy S. Lawrence

**Affiliations:** 1 Entomology and Plant Pathology Department, Auburn University, 559 Devall Dr., Auburn, AL 36849; 2 BASF Corporation, 131 Sapphire Circle, Guyton, GA 31312; 3 Wiregrass Research Center, Headland, AL 36345

**Keywords:** Alabama, *Cannabis sativa*, hemp, root-knot nematode, identification, *Meloidogyne incognita*

## Abstract

Hemp (*Cannabis sativa* L.) is a new crop in Alabama. In 2019, symptomatic plants with stunted growth, poor root development, and numerous galls were observed in hemp plants grown in Geneva County, AL. After harvest, soil samples were collected from areas with the symptomatic plants and root-knot nematode (*Meloidogyne* spp.) were found in the soil. Based on morphological features and the polymerase chain reactions using species-specific primers, it was identified as *Meloidogyne incognita.* Further, a host differential test in a greenhouse assay confirmed it to be *M. incognita* race 3. The pathogenicity of the nematode to the hemp was confirmed by a modified version of Koch’s postulates. To our knowledge, this is the first report of *M. incognita* infecting *Cannabis sativa* in Alabama.

Industrial hemp (*Cannabis sativa* L.) is a new crop for Alabama with the recent legalization and hemp acreage is increasing each year. In the first season of legal production, hemp plants (cultivar ‘Boax’ and ‘Otto2’) in a commercial field located in Geneva County, AL exhibited stunted growth, poor root development, and numerous galls typical of root-knot nematode (*Meloidogyne* spp.) infection. After harvest in September 2019, 75 L of soil were collected from the field in the area with symptomatic plants and four sub-samples were tested for the presence of plant-parasitic nematodes. Nematodes were extracted from the soil by a modified sucrose flotation and centrifugation method (Jenkins, 1964). Root-knot was the only plant-parasitic nematode detected in the soil. The density ranged from 43 to 132 second-stage juveniles (J2) per 500 cm^3^ of soil. Individual juveniles were picked and examined morphologically and molecularly for species identification. The juveniles (*n* = 20) exhibited the following measurements: body length (mean = 402.4 ± 44.0 µm, range = 342.2–457.8 µm), greatest body diameter (14.9 ± 1.3 µm, 13.1–16.9 µm), a (27.0 ± 1.4 µm, 25.3–28.8 µm), c (8.7 ± 0.8 µm, 7.4–10.4 µm), stylet length (12.4 ± 1.9 µm, 9.3–15.8 µm), dorsal esophageal gland orifice to base of stylet (DGO) (2.3 ± 0.6 µm, 1.4–3.7 µm), tail length (46.4 ± 3.2 µm, 42.0–51.1 µm), and hyaline tail length (11.1 ± 1.8 µm, 8.0–13.4 µm). The morphological features of the nematode confirmed it to be a *Meloidogyne* species (Hunt and Handoo, 2009). Polymerase chain reaction (PCR) with species-specific primers identified the distinct species of root-knot nematode. DNA extracts from individual juveniles were prepared in sterile distilled water and the solution was immediately used for PCR (Powers and Harris, 1993). Species-specific primers sets IncK-14F/IncK-14R (*M. incognita* specific) (Randig et al., 2002), Far/Rar (*M. arenaria* specific), and F_jav_/R_jav_ (*M. javanica* specific) (Zijlstra et al., 2000) were all used for PCR analysis. The PCR products were run on a 1% agarose gel and compared to positive control sample of *M. incognita* DNA that had previously been identified by this research group (Groover et al., 2019). Only the primers set IncK-14F/IncK-14R amplified the product of 400 bp nucleotide fragment confirming the population to be *M. incognita* (Randig et al., 2002). A single population of *M. incognita* may further be composed of four different physiological races based on their responses to a set of six different hosts (Hartman and Sasser, 1985). To specify the race, the soil samples were mixed and distributed in 500 cm^3^ polystyrene cups. The cups were planted with cotton (*Gossypium hirsutum* L., ‘DP 1646’), tobacco (*Nicotiana tabacum* L., “NC 91’), pepper (*Capsicum annuum* L., ‘CA Wonder’), watermelon (*Citrullus lanatus* (Thunb.) Matsum. & Nakai, ‘Charleston Grey’), peanut (*Arachis hypogaea* L., ‘GA 06 G’), and tomato (*Solanum lycopersicum* L., ‘Rutgers’) as suggested by Hartmann and Sasser (1985) plus two more host crops, corn (*Zea mays* L., ‘Pioneer P1197YHR’) and soybean (*Glycine max* (L.) Merr., ‘William 82’). Each crops was planted with five replicates each. The test was run in November, 2019 and repeated again in January, 2020. After 45 days of growth in a greenhouse at 25 ± 3°C, nematode eggs were extracted from the roots using 0.6% NaOCl (Hussey and Barker, 1973) and then enumerated. The nematode population was well established in roots of tomato (Reproduction factor (RF)  =  7.5), pepper (RF  =  6.9), watermelon (RF  =  6.4), cotton (RF  =  6.3), corn (RF  =  3.4), and soybean (RF  =  2.6) compared to tobacco (RF  =  0.3) and peanut (RF < 0.1). The results demonstrated that tomato, pepper, watermelon, cotton, corn, and soybean were good hosts, but tobacco and peanut were not a suitable host for the *Meloidogyne* population indicating the nematode population as *M. incognita* race 3 ( Hartman and Sasser, 1985).

After confirmation of the nematode species, a greenhouse test was conducted to preform a modified version of Koch’s postulates in a greenhouse pathogenicity test on hemp. Two-week-old seedlings of the hemp cultivar ‘Maverick’ were transplanted into 500 cm^3^ polystyrene pots containing 1:1 mixture of pasteurized field soil and sand, placed in a randomized complete block design with five replications. Each cup was inoculated with 2,500 eggs from the original *M. incognita* population. Non-inoculated cups (*n*  =  5) were used as control and the entire test was repeated. The test was terminated at 45 days after inoculation. At termination, small galls were observed on inoculated plants resulting nematode reproduction factors of 2.1 for the cultivar ‘Maverick.’ Control plants did not exhibit any symptomatic galls. The morphological and molecular characteristics of the root-knot nematodes were similar to the original population. Results of the study suggested that hemp is a good host for *M. incognita*.

Hemp is a ‘new’ crop in the US and its complete pathogens range is yet to be fully understood. Some studies have reported the hemp to have nematicidal effects (Kayani et al., 2012; McPartland and Glass, 2001; Mukhtar et al., 2013); however, various *Meloidogyne* species such as *M. incognita* (McPartland, 1996), *M. javanica* (Pofu et al., 2010; Song et al., 2017), and *M. enterolobii* (Ren et al., 2020) have been reported from different regions of the world to cause significant damage to the hemp. Recent studies within the states have also exhibited that hemp cultivars screened in the greenhouse have varying degrees of susceptibility towards *M. incognita* (Coburn and Desaeger, 2020) and a few are resistant (Hansen et al., 2020). *Meloidogyne incognita* is commonly found in Alabama and has been reported in 46 out of Alabama’s 67 counties. Potential growers of industrial hemp should consider nematode management when developing their pest management plan. To our knowledge, this is the first report of *M. incognita* infecting *Cannabis sativa* in Alabama.
